# Apigenin suppresses PD-L1 expression in melanoma and host dendritic cells to elicit synergistic therapeutic effects

**DOI:** 10.1186/s13046-018-0929-6

**Published:** 2018-10-29

**Authors:** Lu Xu, Yang Zhang, Kang Tian, Xi Chen, Rongxin Zhang, Xindi Mu, Yueguang Wu, Duchuang Wang, Shanshan Wang, Fang Liu, Taishu Wang, Jinrui Zhang, Shuyan Liu, Yingqiu Zhang, Caixia Tu, Han Liu

**Affiliations:** 1 The Second Affiliated Hospital, Institute of Cancer Stem Cell, Dalian Medical University, Dalian, China; 2 Department of Dermatology, Second Affiliated Hospital, Dalian Medical University, Dalian, China; 3 Cancer Biotherapy & Translational Medicine Center of Liaoning Province, Dalian Medical University, Dalian, China; 4 Department of Orthopaedics, First Affiliated Hospital, Dalian Medical University, Dalian, China

**Keywords:** Melanoma, PD-L1, CD274, Apigenin, Flavonoid, STAT1

## Abstract

**Background:**

The PD-L1/PD-1 pathway blockade-mediated immune therapy has shown promising efficacy in the treatment of multiple cancers including melanoma. The present study investigated the effects of the flavonoid apigenin on the PD-L1 expression and the tumorigenesis of melanoma.

**Methods:**

The influence of flavonoids on melanoma cell growth and apoptosis was investigated using cell proliferation and flow cytometric analyses. The differential IFN-γ-induced PD-L1 expression and STAT1 activation were examined in curcumin and apigenin-treated melanoma cells using immunoblotting or immunofluorescence assays. The effects of flavonoid treatment on melanoma sensitivity towards T cells were investigated using Jurkat cell killing, cytotoxicity, cell viability, and IL-2 secretion assays. Melanoma xenograft mouse model was used to assess the impact of flavonoids on tumorigenesis in vivo. Human peripheral blood mononuclear cells were used to examine the influence of flavonoids on PD-L1 expression in dendritic cells and cytotoxicity of cocultured cytokine-induced killer cells by cell killing assays.

**Results:**

Curcumin and apigenin showed growth-suppressive and pro-apoptotic effects on melanoma cells. The IFN-γ-induced PD-L1 upregulation was significantly inhibited by flavonoids, especially apigenin, with correlated reductions in STAT1 phosphorylation. Apigenin-treated A375 cells exhibited increased sensitivity towards T cell-mediated killing. Apigenin also strongly inhibited A375 melanoma xenograft growth in vivo, with enhanced T cell infiltration into tumor tissues. PD-L1 expression in dendritic cells was reduced by apigenin, which potentiated the cytotoxicity of cocultured cytokine-induced killer cells against melanoma cells.

**Conclusions:**

Apigenin restricted melanoma growth through multiple mechanisms, among which its suppression of PD-L1 expression exerted a dual effect via regulating both tumor and antigen presenting cells. Our findings provide novel insights into the anticancer effects of apigenin and might have potential clinical implications.

## Background

Melanoma is a common type of skin cancer that is frequently associated with poor clinical outcomes. Targeted therapies against activating mutations occurring in *BRAF* have significantly prolonged patient survivals, although about 50–60% of melanoma patients lack such mutations and thus are not applicable for BRAF tyrosine kinase inhibitor-based treatment [1–3]. Nonetheless, recent advances in immunotherapy have provided exciting improvements in the clinical treatment of melanoma, wherein the immune checkpoint blockade mediated by PD-1/PD-L1 antibodies reactivated immune killing of melanoma cells [4, 5]. Taking its advantages of high immunogenicity and the abundance of adjacent immune cells, melanoma has become a successful leading example of immune checkpoint blockade-based immunotherapy, proving the PD-1/PD-L1 pathway as a top therapeutic target in this skin malignancy [6, 7].

Programmed cell death ligand-1 (PD-L1), also known as B7-H1 and CD274, functions by interacting with its cognate receptor programmed cell death-1 (PD-1) to negatively regulate T cell functions, and therefore plays a pivotal role in the immune evasion of many cancer types [6, 8]. PD-L1 expression is frequently detected in tumor cells and tumor-associated antigen-presenting cells (APCs), including dendritic cells (DCs) and macrophages, which recognizes PD-1 receptor expressed on T cell surface to cause immune suppression [7, 9]. Monoclonal antibodies targeting PD-1, such as nivolumab and pembrolizumab, and the PD-L1 antibody atezolizumab effectively block the PD-1/PD-L1 interaction, representing a successful approach of immune checkpoint blockade that has received multiple FDA approvals in cancer treatment [10, 11].

Epidemiological studies have reported an inverse association between the dietary intake of flavonoids and the risk of cancer [12]. Apigenin is a naturally occurring flavonoid that can be found in many fruits and vegetables. Accumulating evidence has revealed the anti-inflammatory, anti-oxidant, and anti-cancer characteristics of this flavonoid [13–15]. Regarding the anti-cancer properties of apigenin, it has been shown to cause cell cycle arrest and induce the apoptosis of multiple types of malignancies including melanoma [16–21]. However, the effects of apigenin on the PD-1/PD-L1 checkpoint and resultant immune response towards cancer remain underexplored till now.

In the present study, we carefully examined the anti-tumor and immunomodulatory activities of apigenin towards melanoma using both in vitro and in vivo assays. In addition to confirming the growth-suppressive and pro-apoptotic functions of apigenin against melanoma cells, our observations revealed that apigenin was capable of stimulating immune responses towards melanoma cells in vivo, through restricting PD-L1 expression in both melanoma and dendritic cells. Therefore, our findings disclosed another facet of the inhibitory effects of apigenin towards melanoma, which might have potential clinical implications.

## Methods

### Cell culture

The human melanoma cell lines (A375, A2058, and RPMI-7951) and Jurkat cells were obtained from the American Type Culture Collection (Manassas, VA, USA). A375 and A2058 cells were maintained in Dulbecco’s modified Eagle’s medium (DMEM, Gibco, USA), RPMI-7951 cells were maintained in Eagle’s Minimum Essential Medium (EMEM, Gibco, USA), and Jurkat cells were cultured in RPMI 1640 medium (Gibco, USA). All cell culture media were supplemented with 10% fetal bovine serum (ExCell Bio, Shanghai) and 1% penicillin/streptomycin (Thermo Fisher Scientific). All cells were cultured in a humidified incubator with 5% CO_2_ at 37 °C.

### Melanocyte isolation

The experimental procedures were approved by the Ethics Committee of Dalian Medical University and written consent forms were obtained from the participants. The foreskin samples were obtained from circumcision operations performed at the Department of Urology in the Second Affiliated Hospital of Dalian Medical University. The subcutaneous fat tissue of foreskin was gently removed with scissors. Remaining tissue samples were dissected into small pieces (0.5 × 0.5 cm) and incubated in 2.4 mg/mL Dispase II neutral protease (Roche, Mannheim, Germany) at 37 °C for 2 h. The epidermis was dissected from dermis and incubated with 0.25% trypsin at 37 °C for 10 min. Pieces of epidermis were filtered through a 200 mesh screen to collect single cells. The melanocytes were then seeded into flasks and maintained in M254 medium (Gibco, USA) with human melanocyte growth supplements (HMGS; Cascade Biologicals, Portland, Oregon) and 1% penicillin/streptomycin (Thermo Fisher Scientific).

### Antibodies and other reagents

Rabbit anti-PD-L1 (13684, 1: 1000), rabbit anti-phospho-STAT1 (Tyr701) (7649, 1: 1000), and mouse anti-total STAT1 (9176, 1: 1000) antibodies were purchased from Cell Signaling Technology. Rabbit anti-PD-L1 (EPR20529, 1: 1000) were from Abcam. Mouse anti-CD274 (B7-H1, PD-L1) (329709, 1: 50) antibody was from BioLegend. Mouse anti-GAPDH (60004–1, 1: 5000) antibody was obtained from Proteintech. Recombinant human interferon-γ (IFN-γ) was from T&L Biotechnology. Apigenin (HY-N1201) and curcumin (HY-N0005) were purchased from MCE.

### Cell viability assay

The proliferation of melanoma cells was determined by MTT (3-[4,5-dimethylthiazol-2-yl]-2,5-diphenyltetrazolium bromide tetrazolium) assay as described previously [22]. In brief, cells were plated at a density of 2000 cells/well in 96-well plates and incubated overnight. Next day, cells were treated with apigenin, curcumin, or DMSO as control at indicated concentrations for 24 h. To examine cell proliferation, 20 μL of MTT solution (5 mg/ml) was added to each well. Then DMSO was added to dissolve formazan and the absorbance was recorded at 570 nm and 630 nm with a spectrometer.

### Flow cytometric analysis

Cell cycle analyses were carried out as described previously [23]. Briefly, cells were treated with DMSO (control), apigenin, or curcumin for 24 h, prior to harvest and fixation in ice-cold 70% ethanol for overnight at 4 °C. Cells were incubated in PBS buffer containing 100 μg/mL of RNase, 50 μg/mL of propidium iodide (PI), and 0.2% (*w*/*v*) of Triton X-100 for 15 min at room temperature in the dark, before final analysis by flow cytometry. For the analysis of apoptosis, cells were treated with DMSO (control), apigenin, or curcumin for 24 h. Then samples were processed for Annexin V and PI double-staining with an apoptosis assay kit (KeyGEN Biotech, China) as per the manufacturer’s instructions. For the detection of cell surface PD-L1, cells were suspended in PBS/B/A buffer (PBS plus 0.1% BSA and 0.02% NaN_3_) and incubated with PE-conjugated mouse IgG1κ (12–4714-42, eBioscience) or PE-conjugated anti-human PD-L1 antibody (12–5983-42, eBioscience) on ice for 30 min. After PBS washes, samples were resuspended in PBS/B/A buffer and analyzed by flow cytometry. For the analysis of CD4^+^ and CD8^+^ T lymphocytes in mice, excised spleens were gently grinded and filtered through 30 μm nylon screens. Single-cell suspensions were incubated with RBC lysis buffer for 5 min at room temperature. Splenocyte samples were blocked with the CD16/CD32 (14–0161-81, eBioscience) antibody and then stained with the FITC-conjugated CD4 (11–0041-81, eBioscience) and APC-conjugated CD8 (100712, Biolgend) antibodies on ice for 30 min. Each sample was washed twice and finally resuspended in 500 μl of PBS buffer. All samples were analyzed by flow cytometry using a benchtop Accuri C6 cytometer (BD Biosciences). Flow cytometry data were finally analyzed using FlowJo version 7.6.1 (FlowJo, LLC, USA).

### Cell lysis and Western blotting

Cells were lysed on ice in RIPA lysis buffer (10 mM Tris-HCl pH 7.5, 100 mM NaCl, 50 mM NaF, 1% *w*/*v* NP-40, 0.1% w/v SDS, 1% sodium deoxycholate) containing mammalian protease and phosphatase inhibitors (Sigma). Cell lysates were centrifuged at 20, 000 x g for 30 min to remove debris. Protein concentrations were measured by the Bradford assay. Equal amounts of protein samples were separated by SDS-PAGE and protein bands were transferred to a nitrocellulose membrane (Merck Millipore, USA). Membranes were blocked with 4% fat-free milk in PBS for 1 h at room temperature and then incubated with indicated primary antibodies with gentle shaking at 4 °C for overnight. Membranes were washed with PBS for three times and incubated with corresponding secondary antibodies (LICOR 680 nm or 800 nm infrared-labeled). Image acquisition and quantitation of band intensity were performed using the Odyssey infrared imaging system (LI-COR Biosciences, Lincoln, NE, USA).

### Immunofluorescence

Immunofluorescence was performed as previously described [24]. Cells were seeded onto glass coverslips and cultured in 6-well plates. Cells were washed with PBS twice, fixed with 4% paraformaldehyde for 15 min, permeabilized with 0.2% Triton X-100 for 5 min, and blocked with 5% BSA in PBS for 30 min at room temperature. Samples were stained with primary antibodies for 1 h at room temperature, followed by secondary antibodies at room temperature for 30 min. Nucleus was stained with 4′, 6-diamidino-2- phenylindole (DAPI, Life Technologies). A fluorescent microscope (Olympus, Japan) was used for image acquisition.

### Co-culture experiments and cytokine measurement

A375 cells were pretreated with DMSO (control), 25 μM of curcumin, or 30 μM of apigenin for 4 h and then cultured in the presence of 10 ng/ml of IFN-γ for 24 h. Cells were collected, washed, resuspended and 2 × 10^4^ cells/well were plated into a 12-well flat bottom plate. Melanoma cells were incubated at 37 °C in a humidified CO_2_ incubator for overnight to allow attachment. Jurkat cells were infected with control or PD-1-expressing lentiviruses (GeneChem, Shanghai). Cell surface PD-1 expression was confirmed by FACS analysis. Jurkat cells were activated with 100 ng/mL of CD3 antibody (317303, BioLegend) and 100 ng/mL of CD28 antibody (302913, BioLegend), before co-culture with A375 cells for 72 h. Plates were washed with PBS and the remaining tumor cells were stained with crystal violet solution. The dried plates were scanned and staining intensity was quantified. A cytotoxicity LDH assay kit (Dojindo, Japan) was used to measure the cytotoxic activity on target cells according to the manufacturer’s instructions. Cell-free supernatants were collected and secreted IL-2 in the media were measured as per the manufacturer’s instructions using an IL-2 ELISA Kit (VAL110, R&D Systems).

### Immunohistochemistry

Melanoma samples were fixed in 4% paraformaldehyde and then embedded in paraffin. Tissue sections (5 μm) were processed for the staining of Ki-67 (RMA-0731, Maxim), CD4 (RMA-0514, Maxim), CD8 (RMA-0620, Maxim), and detection was completed using the Streptavidin-Peroxidase IHC assay kit (ZSGB-bio, China) as per the manufacturer’s instructions. Immunohistochemical images were captured using a Leica microscope. The numbers of Ki-67, CD4, and CD8 positive cells in tumor sections were counted from 10 randomly chosen fields at 40 x magnification.

### Xenograft tumor models

Animal experiments were performed in accordance with the 1996 National Institutes of Health Guide for the Care and use of Laboratory Animals, and the procedures were approved by the Institutional Animal Care and Use Committee of the Dalian Medical University. Female C57BL/6 mice (4–6 weeks) were obtained from the Animal Center of Dalian Medical University and maintained under sterile conditions during the entire experiments. To generate tumor xenografts, 2 × 10^5^ of B16-F10 cells in 0.1 mL of PBS were injected subcutaneously into the right flank of each mouse. Tumor-bearing mice were randomly divided into three groups (*n* = 8 per group). Curcumin (50 mg/kg) or apigenin (150 mg/kg) were administrated by oral gavage once a day, and control mice were given the same volume of saline for 12 days. Mice were sacrificed after day 16. Tumor volumes were calculated according to the following formula: tumor volume (mm^3^) = (length) × (width)^2^ × 0.5, and the tumor weights were recorded.

### Isolation of mouse dendritic cells

Single splenic cell suspensions were prepared by grinding the mouse spleens through nylon mesh. After the lysis of red blood cells, splenocytes were incubated with CD11c MicroBeads (130–052-001, Miltenyi Biotec) for 15 min. After washes, CD11c + dendritic cells were isolated by positive selection using MACS separation columns (Miltenyi Biotec, Auburn, CA) according to the manufacturer’s instructions.

### Generation of CIK and DC-CIK

The experimental procedures were approved by the Ethics Committee of Dalian Medical University and written consent forms were obtained from each volunteer participant. Peripheral blood mononuclear cells (PBMCs) were isolated by Ficoll density gradient centrifugation from freshly drawn peripheral blood (50 mL). Cells were allowed to adhere in plastic cell culture flask for 2 h at 37 °C in the incubator. Adherent cells were induced into DCs using 500 U/mL of recombinant human interleukin-4 (PeproTech) and 1000 U/mL of recombinant human granulocyte-macrophage colony stimulating factor (PeproTech) for 4 days, before treated with 1000 U/mL of tumor necrosis factor alpha (PeproTech) to generate mature DCs. Cells were then treated with DMSO (control), curcumin (25 μM), or apigenin (30 μM). Non-adherent cells were collected for cytokine-induced killer (CIK) cells using serum-free medium (Lonza, X-VIVO 15) supplemented with 1000 U/mL IFN-γ (Chemo Wanbang, China) for 24 h. Then cells were treated with 100 ng/mL of anti-CD3 antibody and 1000 U/mL of recombinant human IL-2 (Sanyao, China). The medium was replenished every 3 days. At day 7, CIK cells were harvested and separated into equal parts for co-culture with previously treated DCs at a ratio of 3:1 for another 7 days. CIK and DC-CIK cells were used in co-culture assays with A375 cells according to methods described above.

### Cytotoxicity assay

A375 cells were pretreated with DMSO (control), 25 μM of curcumin, or 30 μM of apigenin for 4 h and then cultured in the presence of 10 ng/mL of IFN-γ for 24 h. Cells were then distributed into 96-well plates at a density of 2 × 10^4^ cells/well in triplicates, with an effector (CIK, DC-CIK, DC-APG-CIK, and DC-CCM-CIK) to target ratio of 10:1 for 20 h. The supernatant was collected from each well and the activity of released LDH was determined using a cytotoxicity LDH assay kit (Dojindo, Japan) according to manufacturer’s instructions. Cytotoxicity was calculated as follows: cytotoxicity (%) = (test substance - low control)/(high control - low control) × 100.

### Statistical analysis

Data are presented as mean ± standard deviation (S.D.) from at least 3 biologically repeated experiments. Two-tailed Student’s t-tests were performed to compare the statistical differences between treatment groups using GraphPad Prism software (version 7), *p* < 0.05 was considered statistically significant.

## Results

### Growth-suppressive and pro-apoptotic effects of flavonoids

**Fig. 1:**
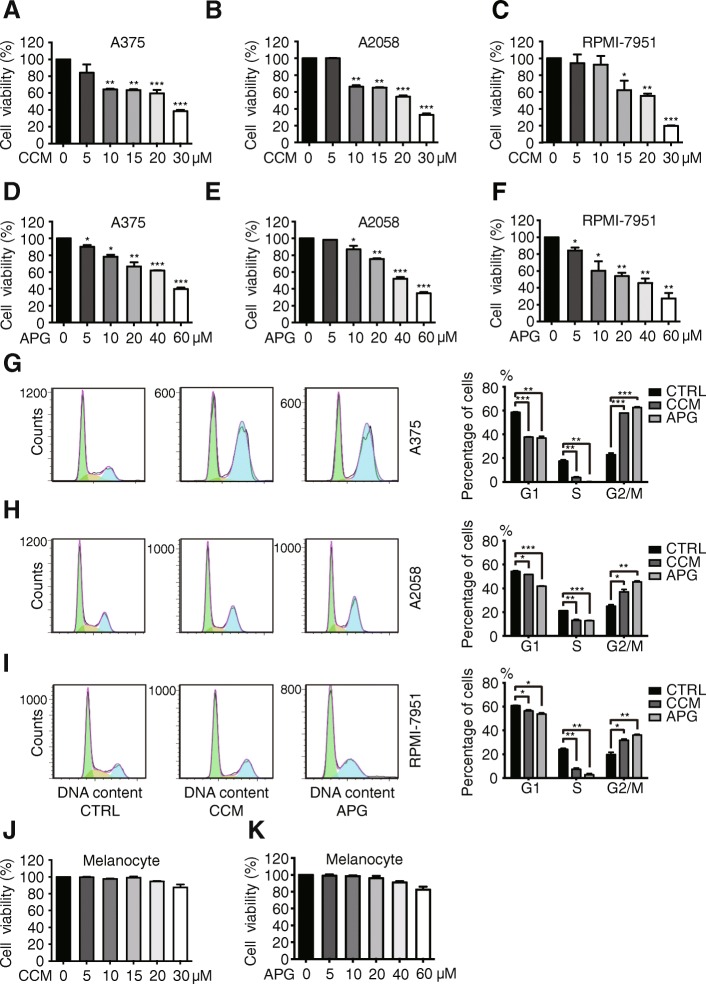
Apigenin and curcumin induce cell cycle blockage to inhibit melanoma cell growth. **a**-**f** A375, A2058, and RPMI-7951 melanoma cells were treated with apigenin or curcumin at various concentrations for 24 h and cell viability was detected by MTT assays. **g**-**i** indicated melanoma cells were treated with DMSO as control, curcumin (CCM, 25 μM), or apigenin (APG, 30 μM) for 24 h and cell cycle distribution was analyzed by flow cytometry. The column charts on the right show the quantitation of cells distributed at each stage in percentage. **j**-**k** primary melanocytes were treated with curcumin and apigenin at indicated concentrations for 24 h and cell viability was measured by MTT assays. Data were presented as the mean ± S.D. from three independent experiments. *P < 0.05, **P < 0.01, ***P < 0.001

The anti-cancer effects of flavonoids including apigenin and curcumin have been reported previously [25, 26]. To confirm such impact of apigenin and curcumin in our melanoma cell systems, we performed a series of assays to examine the influence of apigenin and curcumin on the cell proliferation and apoptosis of A375, A2058, and RPMI-7951 melanoma cells. As illustrated in Fig. 1a-f, both curcumin and apigenin effectively suppressed the propagation of all 3 melanoma cell lines, in a concentration-dependent manner. Curcumin treatment elicited a stronger inhibition compared to apigenin, with a concentration at 30 μM of curcumin showing similar effects as caused by 60 μM of apigenin. In the following flow cytometric analyses of cell cycle distribution, both curcumin and apigenin treatment led to cell cycle blockage at G2/M phases, with concurrent decreased cell numbers distributed at G1 and S phases in A375, A2058, and RPMI-7951 cells (Fig. 1g-i). On the contrary, normal primary melanocytes appeared to be tolerating to both curcumin and apigenin treatment at concentrations up to 30 and 60 μM, respectively (Fig. 1j, k).

**Fig. 2:**
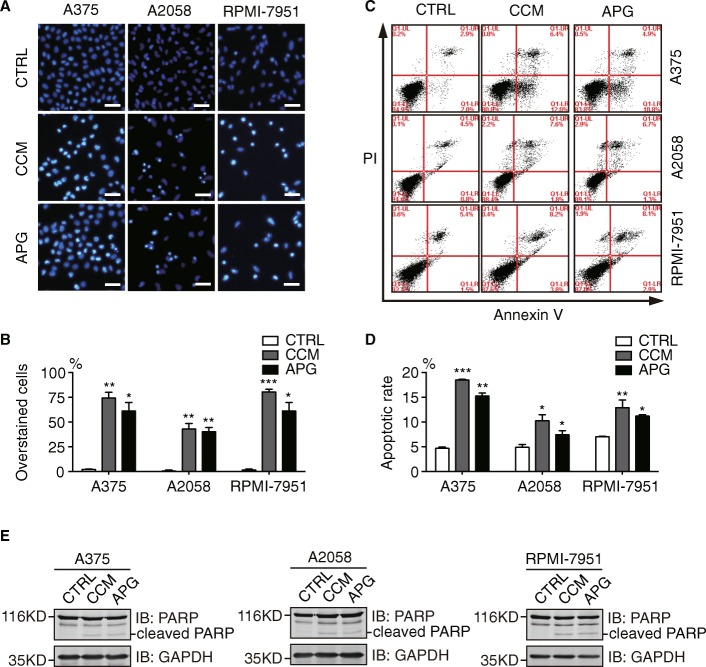
Apigenin and curcumin induce apoptosis in melanoma cells. **a** A375, A2058, and RPMI-7951 cells were treated with DMSO (control), curcumin (25 μM), or apigenin (30 μM) for 24 h before stained with Hoechst 33342. More than 200 cells were counted from 3 random views and percentages for apoptotic cells were shown in (**b**). Scale bar = 50 μm. **c** melanoma cells were stained with Annexin V and PI before flow cytometric analysis following 24 h treatment with DMSO, curcumin (25 μM), or apigenin (30 μM). **d** quantitation data of apoptosis from (**c**). **e** A375, A2058, and RPMI-7951 were treated as mentioned above and cleaved PARP was detected by Western blot analysis. GAPDH was shown as a loading control. All experiments were performed with 3 biological repeats. Error bars represent S.D. *P < 0.05, **P < 0.01, and ***P < 0.001

Next, we examined the pro-apoptotic effects of curcumin and apigenin in A375, A2058, and RPMI-7951 cells. As illustrated in Fig. 2a, b, curcumin and apigenin treatment considerably increased the proportions of Hoechst 33342 overstained cells, indicative of compromised membrane integrity incurred by curcumin and apigenin in all 3 melanoma cell lines. We then carried out flow cytometric analysis to measure the amounts of apoptotic cells in curcumin and apigenin-treated melanoma cells using propidium iodide and Annexin V double staining. Consistently, curcumin and apigenin treatment resulted in significant increases in the percentages of apoptotic populations in A375, A2058, and RPMI-7951 cells (Fig. 2c, d). Furthermore, we detected elevated levels of cleaved PARP proteins from curcumin and apigenin-treated A375, A2058, and RPMI-7951 cell lysates compared to control samples using Western blotting analyses, confirming the pro-apoptotic activities of these flavonoids (Fig. 2e).

### Curcumin and apigenin inhibit IFN-γ-induced PD-L1 expression

**Fig. 3:**
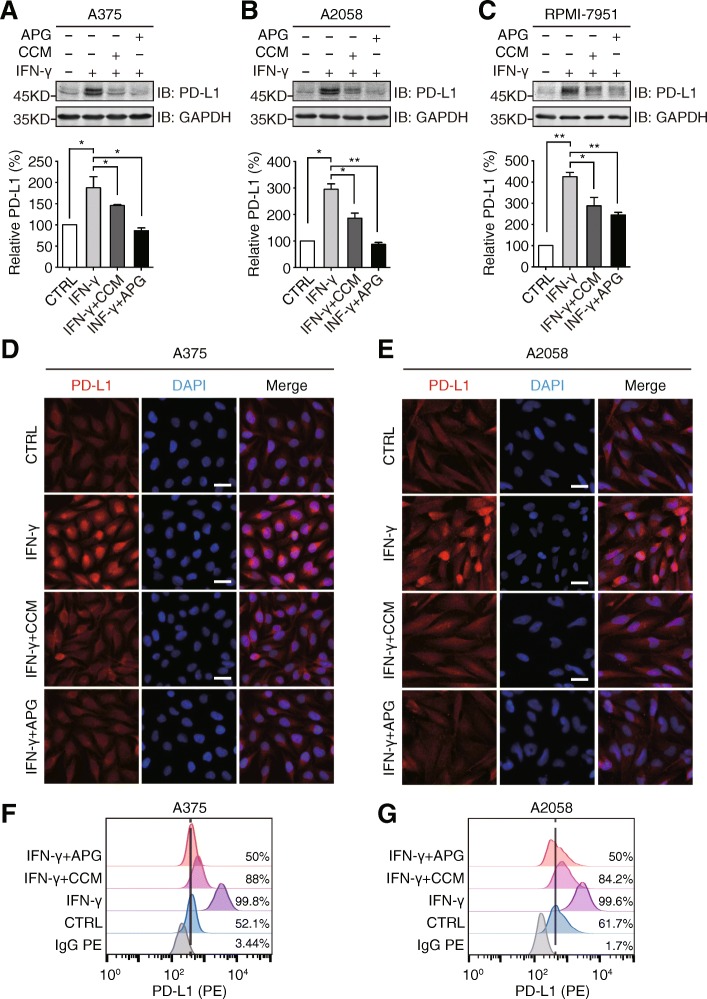
Apigenin and curcumin suppress IFN-γ-induced PD-L1 expression in human melanoma cells. **a**-**c** indicated melanoma cells were pretreated with DMSO, curcumin (25 μM), or apigenin (30 μM) for 4 h and then treated with IFN-γ (10 ng/ml) for 24 h. Cells were lysed and PD-L1 expression was detected by Western blotting using a PD-L1 antibody. GAPDH was used as a loading control. Column charts below show quantitation data of relative PD-L1 expression. Error bars represent the mean ± S.D. (*n* = 3, *P < 0.05 and **P < 0.01). **d** and **e** cells were treated as described above and processed for immunofluorescence analysis. Representative micrographs show PD-L1 staining from different groups with same intensity settings on a fluorescent microscope (Olympus, Japan). DAPI stains nucleus. Scale bar = 25 μm. **f** and **g**, A375 and A2058 cells were treated as described above, and cell surface PD-L1 expression was determined by flow cytometry

Since the influence of flavonoid on the PD-L1 expression in tumor cells so far remains under-investigated, especially in melanoma, we assessed the impact of curcumin and apigenin on the IFN-γ-induced upregulation of PD-L1 expression in A375, A2058, and RPMI-7951 cells. As illustrated in Fig. 3a-c, results from Western blotting experiments showed that IFN-γ addition strongly stimulated the expression levels of PD-L1 in A375, A2058, and RPMI-7951 cells, while both curcumin and apigenin substantially attenuated this PD-L1 upregulation in all 3 cell lines, with apigenin showing a uniformly stronger suppression than curcumin. Subsequently, we performed immunofluorescence assays to examine the influence of curcumin and apigenin on the IFN-γ-induced upregulation of PD-L1 expression in A375 and A2058 cells. In accordance with data from immunoblotting analysis, curcumin and apigenin treatment remarkably reduced the IFN-γ-induced enhancement of the fluorescence signal from PD-L1 staining (Fig. 3d, e). In order to further investigate the impact of both flavonoids, we conducted flow cytometric analyses to assess the PD-L1 levels on melanoma cell surface. As illustrated in Fig. 3f, g, curcumin and apigenin significantly attenuated IFN-γ-induced PD-L1 expression on cell membranes.

**Fig. 4:**
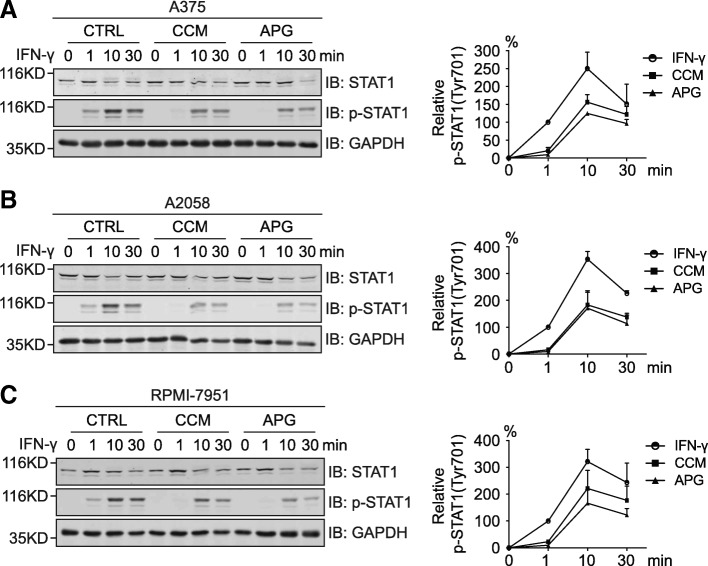
Apigenin and curcumin attenuate IFN-γ-induced STAT1 phosphorylation in human melanoma cells. **a-c**, A375, A2058, and RPMI-7951 cells were incubated with DMSO, curcumin (25 μM), or apigenin (30 μM) for 4 h and then treated with IFN-γ (10 ng/ml) for indicated times. The levels of STAT1 phosphorylation at Tyr701 and total STAT1 were detected by Western blot analysis. GAPDH was used as a loading control. Line charts on the right show the quantitation data of pSTAT1 by comparing to the levels with 1 min IFN-γ stimulation from the control group (100%) of each cell line. Data were presented as the mean ± S.D. from three independent experiments

It has been well-established that the IFN-γ-induced upregulation of PD-L1 expression in tumor cells is mediated transcriptionally through the activation of signal transducer and activator of transcription 1 (STAT1) downstream of the IFN-γ receptor [27]. Therefore, we speculated that curcumin and apigenin might dampen this process through the inhibition of STAT1 phosphorylation. As expected, results from Western blotting analyses revealed that both flavonoids significantly attenuated the acute activation of STAT1 induced by IFN-γ addition (Fig. 4a-c). In all 3 melanoma cell lines, apigenin led to a greater suppression of the phosphorylation of STAT1 compared to curcumin, which was in good correlation with the stronger effect of apigenin observed on the downregulation of PD-L1 expression.

### Curcumin and apigenin enhance T cell-mediated melanoma cell killing

**Fig. 5:**
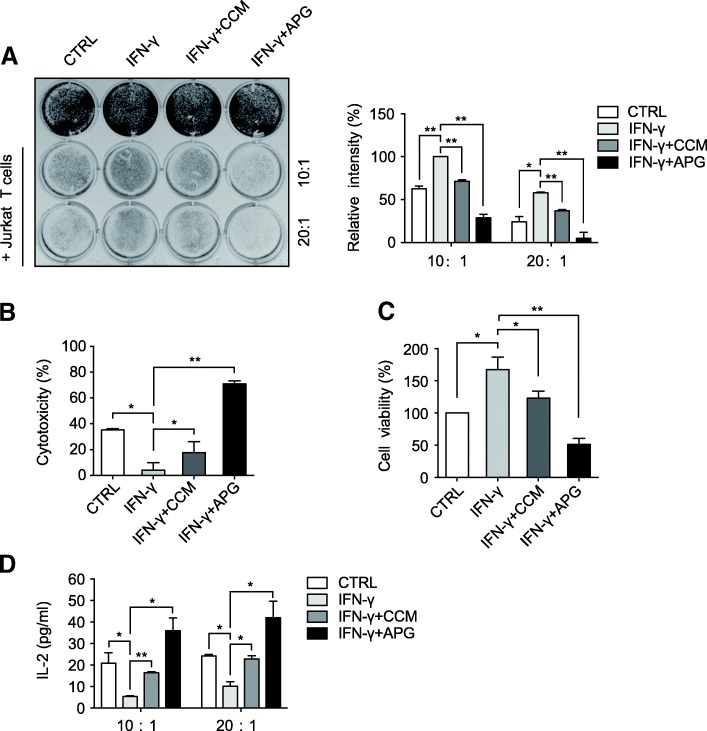
The effects of flavonoids in Jurkat cell-mediated A375 killing assays. **a** A375 cells were treated with DMSO, curcumin, or apigenin in the presence of IFN-γ (10 ng/ml) for 24 h, before cocultured with activated PD-1 expressing Jurkat T cells with effector to target ratios of 10:1 and 20:1. After 72 h of incubation, surviving tumor cells in 12-well plate were stained by crystal violet and imaged. Relative intensities of surviving cells are shown in the right chart, with IFN-γ-treated sample (10:1 group) set to be 100%. **b** and **c** cell killing assays were set up as described above in a 96-well plate. The cytotoxicity of T cells against A375 cells at an effector to target ratio of 10:1 was analyzed using an LDH detection assay kit (percentages calculated as per kit instruction) and remaining cell viability was assessed by MTT assay (control sample set to be 100%). **d** secreted IL-2 from Jurkat-mediated A375 killing assays was measured using supernatants from cocultures by an IL-2 ELISA kit. Data are represented as mean ± S.D. (*n* = 3, *P < 0.05 and **P < 0.01)

Considering the pivotal roles of PD-L1 expression in the immune evasion of cancer cells and our observations on the negative regulation of PD-L1 expression in melanoma cells by curcumin and apigenin, we sought to investigate how these two flavonoids would affect T cell-mediated melanoma cell killing. In doing this, we set up T cell-mediated melanoma cell killing assays using the commonly used A375 melanoma cells and PD-1 expressing Jurkat T cells. Cultured A375 cells were treated with IFN-γ alone or in the presence of curcumin or apigenin, before co-culture with PD-1 expressing Jurkat cells. As illustrated in Fig. 5a, the addition of Jurkat T cells at both 10- and 20-fold excess in cell number of A375 effectively decreased the amounts of surviving melanoma cells, while IFN-γ treatment apparently suppressed T cell killing, resulting in the elevations of remaining A375 cells. In addition, results from T cell killing experiments revealed that flavonoid treatment potentiated T cell-mediated melanoma cell killing, with apigenin exhibiting a potent impact that led to on average 70% and 89% reductions in surviving A375 cells from 10 x and 20 x Jurkat treatment groups, respectively (Fig. 5a).

To confirm the flavonoid-induced enhancement of Jurkat T cell-mediated melanoma cell killing, we employed an alternative approach by measuring the cytotoxicity incurred by Jurkat T cells and resultant cell viability of A375 cells following co-culturing. Cytotoxicity was measured by examined the activity of lactate dehydrogenase (LDH) released into the growth media from damaged cells, while cell viability was determined by a standard MTT assay. As shown in Fig. 5b, IFN-γ treatment considerably reduced cytotoxicity, but the two flavonoids especially apigenin strongly augmented the total activity of LDH released into the media, suggesting severe damages of A375 cells in the apigenin-treated group. Consistently, the measurement of cell viability was in good agreement with results from cytotoxicity assays, wherein the IFN-γ-induced increase (from 100 to 167% on average) in cell viability was dramatically dampened by curcumin and apigenin treatment (from 167 to 123% and 51% on average, respectively) (Fig. 5c). Furthermore, we carried out ELISA assays to measure the levels of IL-2 presented in the growth media of A375 and Jurkat T cell co-cultures, which was an indicator of T cell activity. Results from ELISA analyses showed that the secretion of IL-2 was remarkably suppressed in the IFN-γ-treated group, indicative of compromised T cell activity (Fig. 5d). However, the IL-2 levels in growth media were strongly recovered in the curcumin-treated groups, and were even dramatically elevated in the apigenin-treated samples, suggesting the positive effects of the flavonoids on T cell activity (Fig. 5d). Taken together, our observations suggest that these two flavonoids tested, most prominently apigenin, suppress PD-L1 expression on melanoma cells, thus unleashing PD-L1/PD-1 checkpoint-mediated immune suppression and therefore eliciting more efficient T cell killings.

### Apigenin-mediated suppression of melanoma xenograft growth is associated with increased immune cell infiltration

**Fig. 6:**
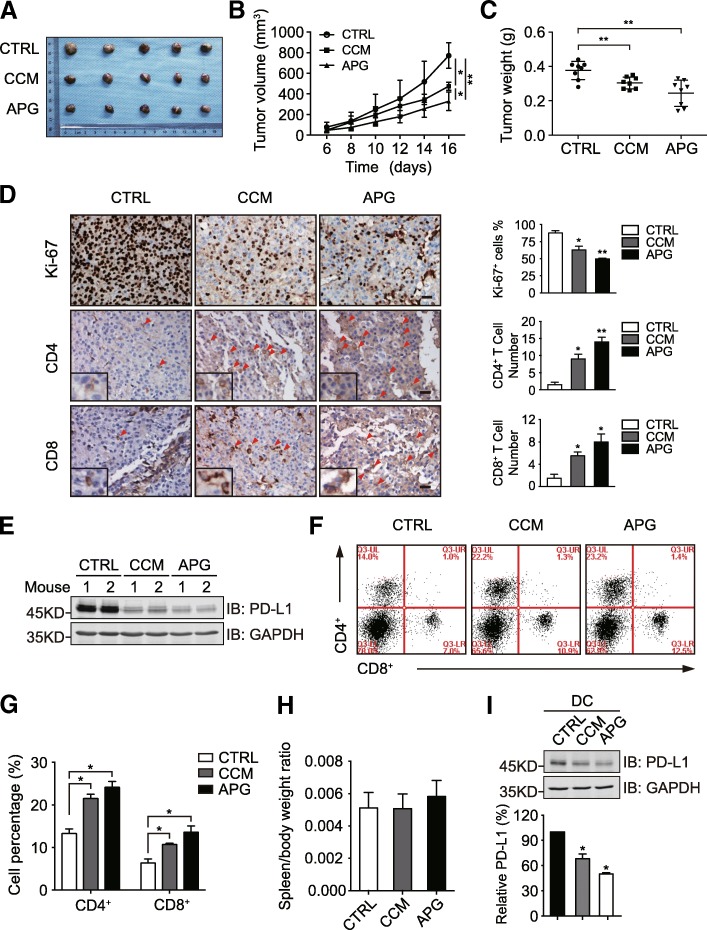
The influence of apigenin on B16-F10 melanoma xenograft tumor growth and host T cell immunity. **a** B16-F10 melanoma cells were subcutaneously inoculated into female C57BL/6 wild-type mice. After 4 days, xenograft-bearing mice were randomly divided into 3 groups to receive saline (control), curcumin, or apigenin by daily oral gavage for 12 consecutive days, before sacrifice and tumor resection. **b** B16-F10 xenograft tumor volume was measured every other day during the treatment and plotted. **c** the weight of resected B16-F10 xenograft tumor was measured and plotted. **d** tumor tissue sections were analyzed by immunohistochemistry using anti-Ki-67, anti-CD4, and anti-CD8 antibodies. Scale bar = 50 μm. Magnified insets show CD4 or CD8 positive stained cells. **e** tumor tissues were lysed to detect PD-L1 expression by immunoblotting. GAPDH was used as a loading control. **f** splenocyte preparations from different groups were analyzed by flow cytometry with CD4 and CD8 antibodies. **g** the quantitation of CD4 and CD8 positive cells from different treatment groups in F (total population as 100%). **h** spleen/body weight ratios of mice with different treatments. **i** C57BL/6 mice with 3 groups were treated by oral gavage with saline, curcumin (50 mg/kg), or apigenin (150 mg/kg) once a day for 2 weeks. DCs were isolated from mouse spleens and PD-L1 expression in DCs was then detected by immunoblotting. Bar chart shows relative quantification of PD-L1 levels (control as 100%). GAPDH was used as a loading control. Data are presented as mean ± S.D. *P < 0.05, **P < 0.01

To investigate the influence of curcumin and apigenin on the tumorigenesis of melanoma in vivo, we generated melanoma xenograft mouse models (wild-type C57BL/6) using the B16-F10 melanoma cell line derived from the C57BL/6 strain. Tumor-bearing mice were randomly divided into 3 groups to receive control saline, curcumin, or apigenin by daily oral gavage. As illustrated in Fig. 6a-c, curcumin and apigenin treatment significantly suppressed the growth of B16-F10 xenograft tumors, with apigenin showing a stronger inhibition than curcumin. We then carried out immunohistochemistry analyses on resected B16-F10 xenograft tumor tissues from different treatment groups. Ki-67 expression was detected to examine the proliferative status of different tumor samples. As shown in Fig. 6d, the percentages of Ki-67 positive cells in curcumin and apigenin-treated groups (62.9 and 49.9%, respectively) were significantly down regulated compared to that from the control group (87.5%). We also investigated the existence of different subpopulations of T cells in xenograft tumor tissues by CD4 and CD8 staining. Interestingly, the numbers of CD4+ and CD8+ cells detected in the tumor tissue sections from curcumin and apigenin-treated mice were apparently increased compared to that from control samples, suggesting that both flavonoids stimulated immune cell infiltration into tumor tissues (Fig. 6d). Meanwhile, results from immunoblotting analyses confirmed that PD-L1 expression was reduced in xenograft tissues receiving flavonoid treatment (Fig. 6e). These observations also prompted us to speculate whether curcumin and apigenin treatment regulated T cell immunity through affecting the proportions of CD4+ and CD8+ cells in the lymphocyte populations of mouse hosts. Therefore, we isolated splenocytes from B16-F10 xenograft-bearing mice treated with saline, curcumin, or apigenin and then carried out flow cytometric analyses to examine the percentages of CD4+ and CD8+ lymphocytes in different samples. As described in Fig. 6f-h, no significant difference in the spleen/body weight ratio was detected from mice with different treatments, but the proportions of CD4+ and CD8+ T cells were both significantly upregulated in the splenocyte preparations from mice treated with curcumin and apigenin compared to those in the saline-treated group. Thus, our findings collectively revealed that curcumin and apigenin not only regulated the sensitivity of tumor to immune killing through the suppression of PD-L1 expression on melanoma cells, but also increased the abundance of CD4+ and CD8+ T cells in the tumor xenograft-bearing mouse hosts.

### Apigenin boosts T cell immunity through suppressing PD-L1 expression in DCs

**Fig. 7:**
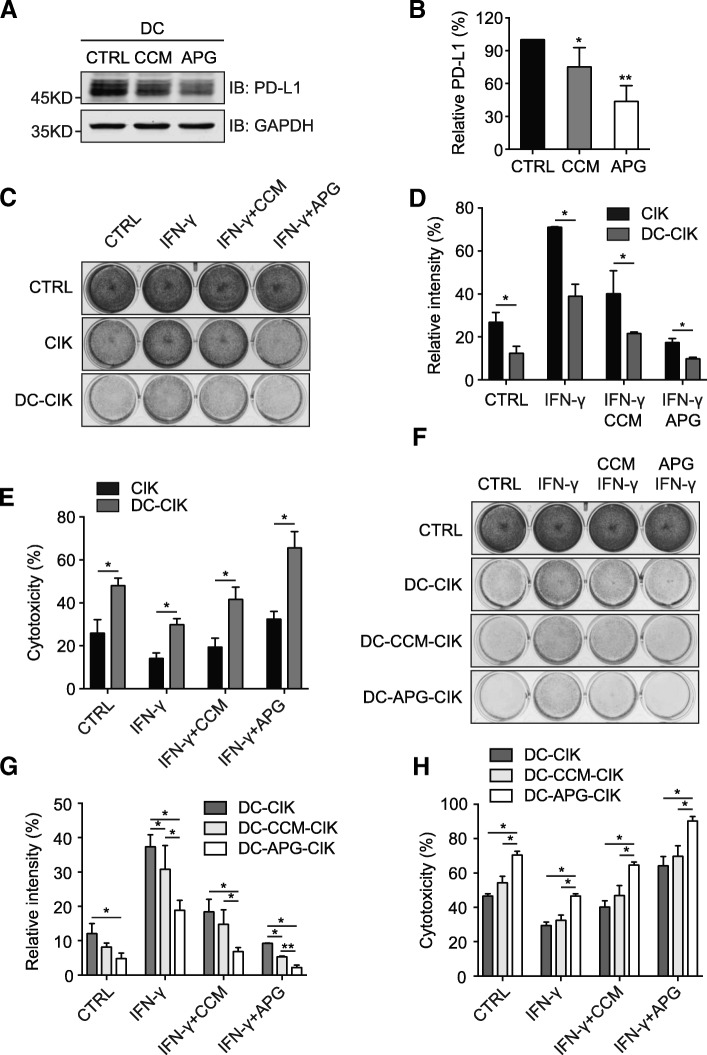
Apigenin decreases PD-L1 expression in DCs to augment host T cell immunity. **a** human PBMC-derived DCs were treated with curcumin or apigenin for 24 h and PD-L1 expression was examined by Western blotting. GAPDH was used as a loading control. **b** quantitation of relative PD-L1 levels from A (control as 100%). **c** A375 killing assays with CIK and DC-CIK cells. Experimental procedures were described in the methods. **d** quantitation of the intensity of surviving A375 cells from C (control set as 100%). **e** cytotoxicity of CIK and DC-CIK cells against A375 as described in C was analyzed using an LDH assay kit. **f** control, curcumin and apigenin-treated DCs were used in cocultures with CIK cells to generate DC-CIK, DC-CCM-CIK, and DC-APG-CIK, respectively, which were then used in A375 killing assays at an effector to target ratio of 10:1 for 20 h. Surviving A375 cells were stained with crystal violet and imaged. **g** quantitation data of relative intensities from surviving A375 cells in F (control set as 100%). **h** cytotoxicity of various DC-CIK cells against A375 was analyzed using an LDH assay kit. Error bars represent S.D. (*n* = 3, *P < 0.05 and **P < 0.01)

Recent advances have provided novel mechanistic insights into the effectiveness of the PD1/PD-L1 pathway blockade-mediated tumor regression, with compelling evidence suggesting that PD-L1 expression in host antigen presenting cells (APCs), including DCs and macrophages, plays pivotal roles in the efficacy of PD1/PD-L1 checkpoint blockade-based immunotherapy [28]. Given that apigenin positively regulated the levels of CD4+ and CD8+ T cells in the melanoma-bearing mouse hosts and this flavonoid was capable of inhibiting the activation of STAT1 following IFN-γ stimulation, we next investigated whether apigenin treatment could influence the PD-L1 expression levels in DCs to regulate T cell immunity against melanoma cells. To this end, we treated normal C57BL/6 mice (4–6 weeks) with 3 groups to receive saline, curcumin, or apigenin by oral gavage for 2 weeks. DCs were isolated from mouse splenocytes, and PD-L1 expression was investigated by immunoblotting analyses. As shown in Fig. 6i, DCs from curcumin- and apigenin-treated mice contained significantly decreased amounts of PD-L1 compared to control samples. Furthermore, we isolated periphery blood mononuclear cells (PBMCs) from freshly drawn blood of 3 volunteer participants, using which adherent monocytes were cultured and induced to form mature DCs while non-adherent cells were grown and activated for cytokine-induced killer (CIK) cells. Matured DCs were treated with curcumin or apigenin, and then cell lysates were analyzed by immunoblotting to detect the levels of PD-L1. As demonstrated in Fig. 7a, b, apigenin strongly suppressed the expression of PD-L1 in DCs, with curcumin also showing moderate inhibition. Subsequently, DCs treated with DMSO, curcumin, or apigenin were used in co-cultures with CIK cells to generate DC-CIK, DC-CCM-CIK, and DC-APG-CIK cells, respectively. As illustrated in Fig. 7c-e, compared to CIK, DC-CIK cells in general showed enhanced cytotoxicity towards untreated A375 cells and those treated with IFN-γ alone or together with flavonoids. Notably, CIK cells co-cultured with apigenin-treated DCs (DC-APG-CIK) exhibited the strongest immunity in the CIK-mediated melanoma cell killing assays, compared to control and curcumin-treated DC co-cultured CIKs (DC-CIK and DC-CCM-CIK, respectively) (Fig. 7f-h). Taken together, these observations revealed the capability of apigenin to augment T cell immunity through restricting PD-L1 expression in DCs.

## Discussion

The PD-1/PD-L1 pathway blockade based immunotherapy represents a paradigm-shifting and groundbreaking therapeutic approach, which has been proved effective in the treatment of multiple types of cancer and thus has received a number of FDA approvals for clinical applications [11, 29]. These immune checkpoint-targeted therapies are mainly based on monoclonal antibodies against PD-1 and PD-L1 that block their interaction. It has been reported that PD-L1 expression in tumor cells and host APCs was associated with tumor immune evasion and poor clinical outcomes [28, 30]. Therefore, targeted therapies that are capable of reducing PD-L1 expression in both tumor cells and APCs might potentially become an alternative therapeutic approach to reactivate host immunity through interfering with the PD-1/PD-L1 immune checkpoint.

Apigenin is a naturally occurring flavonoid that has been reported to possess anti-inflammatory, anticancer, and immune modulating properties [31–33]. In accordance with previous evidence, our observations further confirmed that apigenin, along with curcumin, exhibited strong anti-proliferative and pro-apoptotic effects towards melanoma cells. Interestingly, we also observed an inhibitory effect of apigenin on the expression levels of PD-L1 following IFN-γ stimulation. IFN-γ, a type II interferon, plays critical roles in both innate and adaptive immunity and is secreted from various types of cells including T cells, macrophages, and natural killer (NK) cells [34]. It has been recognized that IFN-γ acts as an important cytokine in tumor microenvironments that can potently induce the expression of PD-L1 in cancer cells to contribute to tumor immune evasion [35, 36]. IFN-γ signaling is transmitted from the IFN-γ receptor to its downstream JAK-STAT1 pathway, wherein activated JAK1 and JAK2 phosphorylate STAT1 on tyrosine 701 to cause the homodimerization of phosphorylated STAT1 [27, 37]. Dimerized STAT1 then translocates into the nucleus and binds to the GAS (IFN-γ-activated sites) elements to initiate a series of transcriptions of IFN-γ-regulated genes, including PD-L1 [27, 38, 39]. Our findings revealed that apigenin strongly suppressed the IFN-γ-induced upregulation of PD-L1 via the inhibition of STAT1 phosphorylation on tyrosine 701, thus providing a mechanistic explanation for its impact on PD-L1 expression in melanoma cells, which was consistent with previous observations of apigenin effect in breast cancer [40].

PD-L1/PD-1 checkpoint-mediated immune suppression is frequently accompanied with reduced IL-2 production, which is a key cytokine required for the proliferation and survival of activated T cells [41]. The blockade of PD-L1/PD-1 interaction has been reported to reactivate T cell functions, with a concomitant increase of IL-2 synthesis and secretion [42, 43]. Similarly, in our T cell-mediated melanoma cell killing assays, apigenin-treated A375 cells displayed enhanced sensitivity towards activated Jurkat T cells, with a correlated increase of secreted IL-2 detected from co-cultures, which indicated that apigenin-incurred PD-L1 downregulation in A375 cells could elicit similar responses as checkpoint blockade.

In order to examine the anticancer effect of apigenin in vivo, we employed a B16-F10 xenograft mouse model with wild-type C57BL/6 genetic background. Notably, apigenin treatment significantly suppressed the growth of xenograft tumors, showing a stronger effect compared to curcumin. It therefore seemed that the immune modulatory functions of apigenin played a more dominant role, since curcumin showed stronger suppressive effects on melanoma cells than apigenin as judged by a series of in vitro assays. Indeed, enhanced T cell infiltration was evident in the melanoma xenograft tissues from apigenin-treated group, which phenomenon resembled those observed from clinical studies with PD-L1/PD-1 pathway blockade [5, 44]. Moreover, the increased tumor T cell infiltration was associated with elevated T cell abundance in the mouse hosts.

**Fig. 8:**
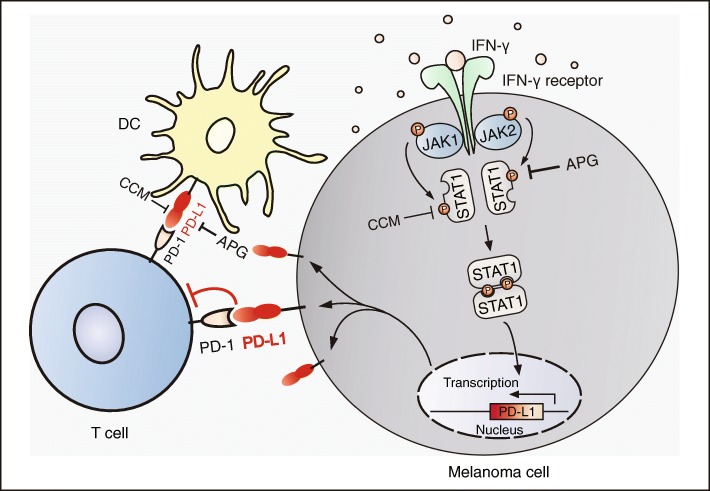
Schematic diagram depicting apigenin and curcumin-mediated inhibition of PD-L1 expression in DCs and melanoma cells. Apigenin and curcumin inhibit IFN-γ-induced STAT1 phosphorylation, leading to reduced PD-L1 expression and surface presentation. Apigenin shows stronger effects than curcumin, with the suppression of PD-L1 expression exerting a dual effect via regulating both tumor and antigen presenting cells (DCs)

To tease out the influence of apigenin on human T cell immunity, we performed further experiments using DCs derived from human PBMCs. Interestingly, apigenin treatment also led to reduced PD-L1 expression in human DCs, which in turn caused an increase in the cytotoxicity of cocultured CIK cells against melanoma. Therefore, our observations revealed the multifaceted features of apigenin in the suppression of melanoma growth, which were exerted either directly through growth inhibition and apoptosis induction, or indirectly but more potently through regulating immune responses in the tumor microenvironment as well as boosting host immunity, both in a PD-L1 expression dependent manner (Fig. 8).

Having demonstrated the potent inhibitory effects of apigenin on melanoma growth, the present study provides further evidence that certain phytochemicals exemplified by apigenin and (−)-epigallocatechin gallate, a major constituent of green tea catechins, can not only serve as cancer chemopreventive agents [45], but also be exploited to develop novel immunotherapeutic strategies in cancer treatment [46]. In addition, our findings also pave the way for further investigations on apigenin to study its efficacy in the combinations with immune checkpoint based therapies, chemotherapeutic agents, targeted therapies, and radiotherapies.

## Conclusions

In the present study, we demonstrated that the flavonoid apigenin strongly suppressed the IFN-γ-induced activation of STAT1, leading to decreased expression levels of PD-L1 in melanoma cells and thus rendering them more sensitive to T cell-mediated killings. On the other hand, apigenin treatment also led to declined expression of PD-L1 in DCs, resulting in enhanced T cell immunity of the host. Hence, apigenin-incurred inhibition on PD-L1 expression manifested a dual effect to restrict melanoma growth, thus providing novel insights into the anticancer effects of apigenin with potential clinical implications.

## Abbreviations

APC: Antigen-presenting cell
APG: Apigenin
CCM: Curcumin
CIK: Cytokine-induced killer
DC: Dendritic cell
DMEM: Dulbecco’s Modified Eagle’s Medium
EMEM: Eagle’s Minimum Essential Medium
IFN-γ: Interferon-γ
IL-2: Interleukin-2
LDH: Lactate dehydrogenase
PBMC: Peripheral blood mononuclear cell
PBS: Phosphate-Buffered Saline
PD-1: Programmed cell death-1
PD-L1: Programmed cell death ligand-1
PI: Propidium iodide
SD: Standard deviation
STAT1: Signal transducer and activator of transcription 1
